# Geoengineering, climate change scepticism and the ‘moral hazard’ argument: an experimental study of UK public perceptions

**DOI:** 10.1098/rsta.2014.0063

**Published:** 2014-12-28

**Authors:** Adam Corner, Nick Pidgeon

**Affiliations:** 1 Climate Outreach & Information Network (COIN), Oxford OX4 1JE, UK; 2 School of Psychology, Cardiff University, Cardiff CF10 3AT, UK; 3 Tyndall Centre for Climate Change Research, Norwich NR4 7TJ, UK

**Keywords:** geoengineering, moral hazard, public perceptions, scepticism, values

## Abstract

Many commentators have expressed concerns that researching and/or developing geoengineering technologies may undermine support for existing climate policies—the so-called moral hazard argument. This argument plays a central role in policy debates about geoengineering. However, there has not yet been a systematic investigation of how members of the public view the moral hazard argument, or whether it impacts on people's beliefs about geoengineering and climate change. In this paper, we describe an online experiment with a representative sample of the UK public, in which participants read one of two arguments (either endorsing or rejecting the idea that geoengineering poses a moral hazard). The argument endorsing the idea of geoengineering as a moral hazard was perceived as more convincing overall. However, people with more sceptical views and those who endorsed ‘self-enhancing’ values were more likely to agree that the prospect of geoengineering would reduce their motivation to make changes in their own behaviour in response to climate change. The findings suggest that geoengineering is likely to pose a moral hazard for *some* people more than others, and the implications for engaging the public are discussed.

## 1. Introduction: the social and ethical dimensions of geoengineering

Slowly but surely the concept of geoengineering—large-scale intentional intervention in the Earth's climatic system to counteract the effects of climate change [1]—has become the focus of both scientific enquiry and policy debate. A range of putative technologies are captured by the term ‘geoengineering’ (which is itself contested—for discussion, see [2]). They share few common features other than their possible utility in either removing carbon dioxide from the atmosphere and sequestering it elsewhere (carbon dioxide removal (CDR) technologies) or reflecting a small proportion of sunlight away from the Earth (solar radiation management (SRM) technologies). CDR technologies include both the biological and chemical capture of carbon dioxide, whereas SRM technologies could operate terrestrially (e.g. via more reflective crops, clouds or infrastructure) or at a stratospheric level (e.g. through the deployment of reflective aerosol particles).

At present, not a single one of the putative technologies that constitute the category ‘geoengineering’ have been developed or tested at the scale required to meaningfully establish their effectiveness or feasibility. However, it is possible to anticipate and reflect on many of the social and ethical issues that the *possibility* of geoengineering the climate raises, in advance of any particular geoengineering technology actually existing [3–7]. In fact, it is the social and ethical concerns that geoengineering seems to present—questions around public consent for research or deployment, how technologies might be governed or the potential for geoengineering to generate conflict, for example—that currently loom larger in policy debates than issues of technical feasibility [8,9].

There is now a small (but rapidly expanding) literature in philosophy and the social sciences that has engaged with complex, inter-related questions around ethics, governance and public engagement [5–7,10–13]. We do not seek to review this here (for an overview, see [11]). Rather, in this paper, we focus on one of the most prominent ethical questions in science and policy debates: whether the prospect of geoengineering will pose a ‘moral hazard’ that undermines political or public support for existing climate policies.

### (a) The moral hazard argument and geoengineering

The concept of a moral hazard is an idea that derives from economics [14]. Where an individual or party is insured against a particular undesired outcome, they may feel protected against the undesired outcome and therefore take greater risks. That is, certain financial arrangements may create perverse incentives for riskier behaviour. For example, a shop owner who is well insured against theft may not be as cautious as an uninsured shop owner would be in securing their premises overnight.

There have been some challenges to the idea that this kind of behavioural adaptation to changing circumstances is inherently a negative outcome, or specifically a *moral* problem [15]. But, importantly, moral hazards are not simply an abstract philosophical concern: they have been empirically documented in multiple decision-making contexts [14]. In terms of individual-level decision-making, health insurance has been linked to the increased performance of unhealthy behaviours [16]. In one striking example at the political level, studies have suggested that humanitarian intervention can lead to an increased expectation of future foreign engagement by rebel groups, thus promoting riskier strategies that increase the overall loss of life [17].

In the context of geoengineering, the moral hazard is not so much an economic risk but a social, ethical and political one. Geoengineering might be perceived as an insurance policy against climate change, undermining support for existing climate policies. In fact, there are multiple levels on which a geoengineering moral hazard might operate. First, the prospect of geoengineering could cause *individuals* to curb voluntary efforts to reduce carbon emissions. The concept of behavioural ‘rebound’ is well understood (the idea that adopting an energy-saving technology can sometimes increase energy use, undermining the overall impact of the technology itself [18]). If geoengineering were perceived by the public as offering a licence to continue carbon-intensive lifestyles, the ‘rebound’ associated with geoengineering technologies could be even more significant. This behavioural version of the problem might be termed an *individual* moral hazard.

Second, people might perceive *others* to be vulnerable to the moral hazard of geoengineering. That is, a particular individual may not believe that geoengineering policies represent a reason for acquiescing on low-carbon commitments, but may see a risk that *others* would modify their attitudes and behaviours. Social norms specifying the urgency of preventative climate policies could shift. This version of the problem might be termed a *social* moral hazard. Finally, at a *political* level, resources might be diverted away from mitigation and adaptation efforts. Rhetorical (and more importantly) financial support for existing climate policies could decline. This version of the problem might be termed a *political* moral hazard. We return to this tripartite division of possible moral hazards when introducing the design of this study.

The implications of a moral hazard effect on existing climate policies would be significant. There is widespread agreement in the scientific community (even among supporters of geoengineering research) that geoengineering technologies could never replace mitigation strategies (only supplement them [1]). Put simply, existing mitigation and adaptation policies are unlikely to be ambitious enough to avert the risks of dangerous climate change [19]. Thus, if the view that geoengineering negated or reduced the need for mitigation were to spread among politicians, the general public, or even scientists themselves, this would pose a serious problem for effective decarbonization. However, there are a range of views on how plausible the dangers of a moral hazard effect really are.

In a review of the ethical issues surrounding geoengineering, Preston [20] argued that the lack of scientific and political discourse on geoengineering prior to 2006 was directly attributable to concern that geoengineering would ‘create a moral hazard that might encourage risky behavior or influence the willingness of parties to engage in mitigation and adaptation’ [20, p. 25]. Thus, there has been an *a priori* assumption among scientists and policy-makers that researching or even discussing geoengineering could distract attention and support from existing climate policies.

It is also clearly the case that previously ‘taboo’ topics in climate policy can rapidly become part of the mainstream: similar concerns to those currently circulating around geoengineering's capacity to distract attention and resources from mitigation policies were expressed with regards to adaptation policies when they were first mooted [21]. Today, adaptation is considered an uncontroversial part of the societal response to climate change. At the very least, a consideration of the trajectory that the adaptation discourse has followed in a relatively short period of time suggests that even contentious issues can quickly become embedded in mainstream policy.

In the most thorough analysis yet published of geoengineering's capacity to pose a moral hazard, Lin [14] concluded that it is likely that geoengineering efforts *will* undermine existing climate policies:

Geoengineering presents a strong economic, political, and psychological temptation to defer difficult and costly actions to future generations. This temptation, whether characterized as moral hazard, risk compensation, or political opportunism, is a serious concern because geoengineering is widely acknowledged to be an inferior, problematic, and at best temporary option for responding to climate risks (p. 711).

However, not all commentators have expressed such pessimistic views. Bunzl [22], for example, argued that the risk of moral hazard is far-fetched and exaggerated, because no serious actors in the scientific or political community view it as anything other than a short-term fix or a supplement to mitigation policies. Hale [23] argues that moral hazard arguments against geoengineering fail for various reasons, one of which is that they are predicated on a counter-factual imaginary of what ‘might have been’—that is, they start from the (unfalsifiable) contention that climate policies *would* have been more ambitious had geoengineering not been part of the discourse. Other commentators—including geoengineering proponents such as David Keith—are more inclined to see the urgency and benefits of conducting research on geoengineering as outweighing the risks associated with moral hazard arguments [24].

Curiously, though, for an argument that hinges so centrally on the *perception* that geoengineering might offer a form of insurance against climate change, there has been very little direct exploration of the moral hazard premise with members of the public. The claim that geoengineering presents a moral hazard is an empirical one, about attitudes, decisions and behaviour [14]. Echoing Hale's concerns about counter-factual falsifiability, Preston [20] has argued that the empirical nature of the moral hazard argument prevents a meaningful assessment being made, because the impact of geoengineering on existing climate policies would only be knowable when (or if) the situation actually unfolded. However, while it may not be possible to know with certainty how the prospect of geoengineering will impact on public attitudes and behaviour, it is possible to explore this question with members of the public now.

### (b) The moral hazard argument and public perceptions

There is only very limited empirical evidence with which to begin to evaluate the perceived risk of a geoengineering moral hazard among the general public. One quantitative study, using a representative sample of US, UK and Canadian opinion and focusing specifically on SRM technologies, found that people were more likely to disagree than agree with the statement ‘Solar radiation management should be used so we can continue to use oil, coal and natural gas’ [25]. In initial focus groups with a small sample of the UK public conducted for the 2009 Royal Society report [1], no support was found for the idea that geoengineering would generate a moral hazard. In fact, the data pointed to a possible ‘galvanizing’ effect of geoengineering: although participants were generally cautious or hostile towards geoengineering proposals, several stated that they would actually be *more* motivated to undertake mitigation actions themselves (such as reducing energy consumption) if they saw government and industry investing in geoengineering research or deployment. This unexpected response seemed to be most pronounced among participants with more sceptical views on climate change.

The question of whether geoengineering might ‘galvanize’ sceptical public opinion is an intriguing one. It is well known that key determinants of climate change scepticism are the values and cultural ‘worldviews’ that people hold [26–28], and that individuals who endorse free-market economic principles and reject the regulation of industry by governments are more likely to be sceptical about climate change. Typically, it is assumed that scepticism about the reality or seriousness of climate change is driven by opposition to the conventional canon of climate policies (which often involve the regulation of polluting industries or restrictions on the freedom of individual behaviour, and thus are threatening to these individuals’ values and worldviews). However, geoengineering is a very different type of climate policy, and one that does not necessarily conflict with the individualistic and economically liberal views of many climate change sceptics.

Experimental work [29] has found that, for individuals who tend to downplay the risks of climate change, learning about geoengineering caused their estimates of climate change risks to increase. The authors argued that because geoengineering represents a policy option that does not conflict with an ‘individualistic’ worldview, the prospect of it may reduce scepticism about climate change among this group of the population. Interestingly, all participants (regardless of their values and worldviews) who were exposed to information about geoengineering were slightly more concerned about climate change risks than those assigned to a control condition. In a UK survey, Bellamy & Hulme [30] found that those with more ‘egalitarian’ worldviews (a set of beliefs revolving around the idea that society should be more equal, and that unregulated markets are a source of this inequality) tended to be most strongly opposed to geoengineering.

Finally, indirect evidence regarding the potential persuasiveness of the moral hazard argument can be derived from the growing number of qualitative studies that have documented a strong preference for mitigation among the UK general public [12,31–33]. A central finding from this body of work is that favourability towards geoengineering is *conditional* on the ambitious pursuit of mitigation policies. That is, most people are not positive towards the idea of geoengineering as a ‘standalone’ policy, and are therefore perhaps unlikely to be susceptible to a moral hazard effect. As Preston [20] has argued, any suggestion of a ‘reverse moral hazard’ effect that galvanizes public opinion is an ‘empirical matter, resolvable only through careful social science research’ [20, p. 25].

However, no study has yet sought to systematically investigate public perceptions of the moral hazard argument. Is it persuasive? What impact does it have on people's views on climate change and geoengineering? And how do people's value orientations and scepticism about climate change interact with their judgements about the moral hazard argument? In this study, we sought to provide an initial answer to these questions.

### (c) The current research

The current research was designed to systematically test public perceptions of the moral hazard argument against geoengineering. Although previous studies have produced findings that indirectly bear on the issue [12,25], no focused investigation of public views on the moral hazard argument has yet been conducted. Specifically, we sought to investigate the following four research questions:

RQ1. How convincing is the moral hazard argument (compared with a counter-argument and a control condition)?
RQ2. How do levels of climate change scepticism and people's underlying values relate to judgements of the moral hazard argument?
RQ3. Do people distinguish between different ‘levels’ of moral hazard (i.e. on their own individual behaviour; others’ views and behaviours; and responses at a political level)?
RQ4. Does consideration of geoengineering *galvanize* support for existing climate policies rather than reduce it?

## 2. Method

### (a) Participants

Six hundred and ten UK participants were recruited using the online market research company Research Now during the summer of 2013. Nationally representative quotas were obtained for key demographic variables including age (*M*=46.45), gender (51% female), socio-economic group (calculated on the basis of the vocation of the chief income earner in their household), ethnicity (92% White; 4% Asian; 2% Black) and geographical region. About 29% indicated that they would vote Labour at the next general election (22% Conservative, 18% UKIP, 7% Lib Dem and 6% Green Party), indicating a spread of political views. About 11% reported that they were a member of an environmental organization.^1^

### (b) Design, materials and procedure

After completing an online consent form, participants answered a series of demographic questions (see participant quotas described above). Next, all participants completed a widely used 12-item scale to measure climate change scepticism (see electronic supplementary material, appendix S1; [34]). All items were rated using a five-point scale from ‘strongly agree’ to ‘strongly disagree’. Two items relating to geoengineering knowledge were included. The first asked whether participants had heard of the term geoengineering previously (‘yes’ or ‘no’). The second asked participants to indicate on a five-point scale how much they knew about geoengineering, from ‘I have not heard about geoengineering’ to ‘I know a great deal about geoengineering’.

Participants were then presented with a screen that was described as containing a ‘factsheet’ titled *Geoengineering: a new approach for tackling climate change?*, ostensibly downloaded from the website climateinfo.org (see electronic supplementary material, appendix S2, for the full factsheets, which were in fact created solely for the purpose of the experiment). Each factsheet contained text describing some of the expected risks of climate change and briefly outlining possible societal responses to it, including mitigation, adaptation and a third (new) option of geoengineering. Thus, geoengineering was situated as one of three options for responding to climate change.

The penultimate paragraph of all versions of the factsheet described geoengineering as follows:

Most geoengineering technologies have not yet been developed, and so there is a great deal of uncertainty about their risks and benefits. There are likely to be some serious risks and side effects associated with geoengineering (e.g. changes to global rainfall patterns) but they are not yet well understood.

The final paragraph of each factsheet contained a brief quote from a (fictional) expert, Professor North (described as a ‘leading atmospheric scientist’). Depending on the experimental condition participants were randomly assigned to, Professor North's ‘quote’ was systematically varied (i.e. the argument manipulation).

We compared the convincingness of the moral hazard argument against geoengineering with an opposing argument: that geoengineering would permit high-carbon lifestyles to continue, and that this would be a positive (rather than undesirable) outcome.

In the *moral hazard* condition, Professor North was reported as saying

My concern is that if we start talking about the possibilities of geoengineering, and how it could be a ‘Plan B’, we will take our eye off the ball with carbon emissions. After all, while geoengineering can treat the symptoms of climate change, it doesn't deal with the underlying cause. Some countries will see geoengineering as an excuse to avoid reducing their own emissions—and that's not fair. We need to focus on existing policies—switching to renewable energy sources, using less energy through changes in people's lifestyles, and regulating industry more effectively.

In the *counter moral hazard* condition, Professor North was quoted as saying

The great thing about geoengineering is that it does not require people to change their behaviour, or for green taxes, or for industry to be more heavily regulated. Sure, there are risks in trying geoengineering, but we can deal with these as they arise. If we could remove carbon dioxide from the atmosphere, or reflect sunlight back into space, we could carry on driving our cars and flying our planes. Geoengineering offers a way of dealing with climate change that doesn't restrict people's freedoms or penalise businesses.

In a *control* condition, included to permit comparison of both arguments against a baseline, Professor North's quote was a rephrased version of some of the information previously included in the factsheet

Geoengineering would involve removing carbon dioxide from the atmosphere and storing it somewhere else (probably underground), or even reflecting a certain amount of sunlight back out into space. There are likely to be some serious risks involved, but at the moment we can say very little with confidence about exactly how geoengineering would work.

Participants were asked to indicate the extent to which they found the argument in Professor North's quote to be convincing (on a five-point scale from ‘very unconvincing’ to ‘very convincing’). In addition to this measure of the argument itself, we included two sets of dependent measures.

The first set captured general views about geoengineering, including a question on geoengineering support (*To what extent would you support geoengineering as a response to climate change?*—measured on a five-point scale from ‘strongly support to ‘strongly oppose’), and two other measures of attitudes towards geoengineering, both recorded on a five-point scale from ‘strongly agree’ to ‘strongly disagree’: *Geoengineering will help the planet more than it will hurt it* and *The risks of geoengineering will outweigh the benefits*.

The second set of dependent variables included four measures specifically designed to capture different aspects of the moral hazard argument (described above). We asked participants to rate the four following statements, on a five-point scale from ‘strongly agree’ to ‘strongly disagree’:

Knowing geoengineering is a possibility makes me feel less inclined to make changes in my own behaviour to tackle climate change (IndividualMoralHazard).

When people find out about geoengineering, it will reduce their motivation to make changes in their own behaviour to tackle climate change (SocialMoralHazard).

If politicians think geoengineering is a possibility, it will make them less likely to pursue other policies to tackle climate change (PoliticsMoralHazard).

The prospect of geoengineering makes me think that the risks of climate change are worse than I thought (GeoGalvanize).

As value orientations play such a central role in public engagement with climate change [26], and based on existing empirical work on perceptions of geoengineering [29], we anticipated that participants' values would also be related to their views on geoengineering. Values are typically defined as ‘guiding principles in the life of a person’ following Schwartz [35], and are measured using a values inventory. Values are arranged into subsets based on extensive previous empirical and theoretical work [35], and the most relevant ones for public engagement with climate change and geoengineering are those that capture ‘self-enhancing’ values and ‘self-transcendent’ values. Self-enhancing values are those that relate to personal achievement, or the accrual of wealth, power or status to oneself. Self-transcendent values, in contrast, are those that relate to the promotion of interests beyond oneself—for example, concern for the welfare of others, the natural environment or an interest in equality. We used four items from the Schwartz [35] values inventory which measure self-transcending values and four items which measure ‘self-enhancing’ values to capture these opposing value orientations (see electronic supplementary material, appendix S1).

When participants had completed all the questions, they read a debriefing screen which informed them of the purpose of the experiment, explained that they had been subject to a minor deception (a fictitious website and ‘expert’ quotation), and provided some background information about the motivation for the study.

The inter-item reliability of each of the multi-item scales in the study was analysed using Cronbach's *α* coefficient. The climate change scepticism score was 0.93 (considered ‘excellent’), the self-transcendent values score was 0.78 (considered ‘acceptable’) and the self-enhancing values score was 0.65 (considered ‘marginally acceptable’ [36]). We therefore included all three scales in subsequent analyses (although the coherence of the self-enhancing values scale was only ‘marginally acceptable’, the extent to which similar scales have been used successfully in previous work [26] gave us confidence that the items could meaningfully be combined).

## 3. Results

About 28% of participants said that they had heard of the term ‘geoengineering’ before. However, only 2% reported that they knew a fair amount or a great deal about geoengineering—a level of knowledge consistent with previous studies [11].

**Figure 1.**
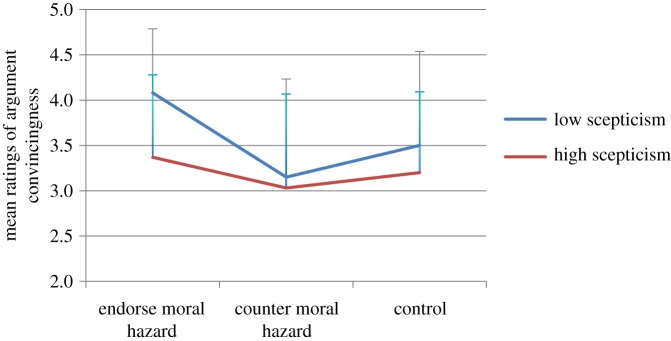
Ratings of argument convincingness in all conditions, by level of scepticism about climate change. Error bars represent 1 s.d. (Online version in colour.)

We first considered the effect of the experimental manipulation and levels of climate change scepticism on ratings of argument convincingness. For these initial analyses, the sample was divided (using a median split) on the basis of their scores on the scepticism scale. Figure 1 shows the mean scores for high- and low-scepticism groups across each of the experimental conditions. A factorial ANOVA revealed a significant effect of the argument-type manipulation, with the *moral hazard* condition more convincing overall than both the control and *counter moral hazard* conditions, *F*_2,604_=22.88, *p*<0.001, eta-squared=0.07. Higher scepticism led to lower ratings of argument convincingness across all three conditions, *F*_1,604_=23.8, *p*<0.001, eta-squared = 0.038. However, there was also a significant interaction between experimental condition and climate change scepticism, *F*_2,604_=4.95, *p*<0.01, eta-squared = 0.016. Simple planned comparisons showed that the difference between high- and low-scepticism groups was significant only in the moral hazard condition, *t*_201_=6.17, *p*<0.001. That is, while scepticism did not determine the convincingness of the *counter moral hazard* argument or control condition argument, the convincingness of the *moral hazard* argument was significantly impacted by levels of climate change scepticism: less sceptical participants were particularly convinced by the *moral hazard* argument.

The three ‘general’ geoengineering-dependent variables were combined into a single measure (Cronbach's *α*=0.64) which we called *GeoGeneral*. A linear regression (with blocks of variables entered sequentially) was conducted on this combined measure in order to determine the impact of the experimental manipulation (dummy coded against the control condition) alongside the other predictor variables included in the study (demographics, climate change scepticism and value orientations^2^). Table 1 displays the results of this regression. The final model accounted for approximately 8% of the variance in *GeoGeneral* scores. The experimental manipulation had no statistically significant effect. However, climate change scepticism scores and age were significant negative predictors of views about geoengineering, whereas only the endorsement of self-enhancing values was a positive significant predictor.

**Table 1.** Blocked linear regression for *GeoGeneral* scores.

	predictor	model 1	model 2	model 3	model 4
	variables	(*β*)	(*β*)	(*β*)	(*β*)
demographics	age	−0.183***	−0.185***	−0.146***	−0.108*
	gender	−0.066	−0.066	−0.073	−0.057
	S.E. group	0.046	0.047	0.057	0.057
experimental group	endorse moral hazard versus control		−0.025	−0.019	−0.025
	counter moral hazard versus control		0.024	0.032	0.029
environmental beliefs	scepticism score			−0.135***	−0.144***
	env. group membership			0.074	0.070
values	self-enhancing				0.174***
	self-transcending				−0.005
adjusted *R*^2^		0.034	0.034	0.054	0.080

***p<0.001, *p<0.05.

We conducted three further regressions to establish the factors that determined participants’ judgements of the three moral hazard-dependent variables (*individual/social/political*). There was a distinct profile for each of these measures—with scepticism playing a central role. In the first regression (*IndividualMoralHazard*), we examined what predicted support for the statement: *Knowing geoengineering is a possibility makes me feel less inclined to make changes in my own behaviour to tackle climate change*. The final model accounted for around 22% of the variance. Higher scepticism, identification with self-enhancing values and higher socio-economic status were all positive, significant predictors of agreement with this statement. Older people and women were more likely to disagree with the *IndividualMoralHazard* argument (table 2).

**Table 2.** Blocked linear regression of factors predicting *IndividualMoralHazard* ratings.

	predictor	model 1	model 2	model 3	model 4
	variables	(*β*)	(*β*)	(*β*)	(*β*)
demographics	age	−0.130***	−0.132***	−0.189***	−0.108***
	gender	−0.111**	−0.111**	−0.087*	−0.047
	S.E. group	0.103*	0.102*	0.096*	0.099*
experimental group	endorse moral hazard versus control		0.034	0.030	0.019
	counter moral hazard versus control		0.053	0.033	0.031
environmental beliefs	scepticism score			0.292***	0.259***
	env. group membership			−0.059	−0.056
values	self-enhancing				0.336***
	self-transcending				−0.071
adjusted *R*^2^		0.033	0.032	0.111	0.219

***p<0.001, **p<0.01, *p<0.05.

In the second regression (*SocialMoralHazard*), we explored the factors predicting agreement with the statement: *When people find out about geoengineering, it will reduce their motivation to make changes in their own behaviour to tackle climate change*. The final model accounted for 7% of the variance in this variable, and there was a consistent effect of the experimental manipulation: participants were more likely to agree that *others* would fall victim to the moral hazard trap if they had read the *moral hazard* argument. Agreement was also positively predicted by being a member of an environmental group and endorsing self-transcending values. Higher scepticism about climate change was a negative predictor of agreement (table 3).

**Table 3.** Blocked linear regression of the factors predicting agreement with *OtherMoralHazard*.

	predictor	model 1	model 2	model 3	model 4
	variables	(*β*)	(*β*)	(*β*)	(*β*)
demographics	age	−0.083*	−0.085*	−0.031	−0.043
	gender	0.011	0.012	0.004	−0.010
	S.E. group	−0.037	−0.037	−0.022	−0.026
experimental group	endorse moral hazard versus control		0.100*	0.109*	0.107*
	counter moral hazard versus control		0.069	0.080	0.065
environmental beliefs	scepticism score			−0.163***	−0.125***
	env. group membership			0.143***	0.134***
values	self-enhancing				0.052
	self-transcending				0.143***
adjusted *R*^2^		0.003	0.008	0.053	0.072

***p<0.001, *p<0.05.

A third distinct profile was observed for agreement with the statement: *If politicians think geoengineering is a possibility, it will make them less likely to pursue other policies to tackle climate change*. The final model accounted for approximately 12% of the variance. Demographic characteristics this time had no effect, and neither did the experimental manipulation. However, environmental group members and those endorsing self-transcendent values were more likely to agree that the possibility of geoengineering would make politicians less likely to pursue other climate change policies (while higher climate change scepticism was associated with lower agreement with this statement; table 4).

**Table 4.** Blocked linear regression for predictors of agreement with *PoliticsMoralHazard*.

	predictor	model 1	model 2	model 3	model 4
	variables	(*β*)	(*β*)	(*β*)	(*β*)
demographics	age	−0.035	−0.036	0.036	0.011
	gender	0.031	0.031	0.019	−0.004
	S.E. group	−0.055	−0.056	−0.036	−0.041
experimental group	endorse moral hazard versus control		0.067	0.079	0.077
	counter moral hazard versus control		0.049	0.064	0.044
environmental beliefs	scepticism score			−0.234***	−0.178***
	env. group membership			0.169***	0.157***
values	self-enhancing				0.031
	self-transcending				0.201***
adjusted *R*^2^		0.000	0.000	0.083	0.118

***p<0.001.

Finally, we analysed *GeoGalvanize* responses and tested the hypothesis that climate sceptics’ interest in climate change might be galvanized by geoengineering. A linear regression accounted for approximately 18% of the variance in GeoGalvanize scores (table 5). Women, younger people and those who more strongly endorsed self-enhancing values were more likely to agree with the statement: *The prospect of geoengineering makes me think that the risks of climate change are worse than I thought*. People scoring more highly on climate change scepticism were significantly *less* likely to agree with this statement (table 5).

**Table 5.** Blocked linear regression of factors predicting agreement with *GeoGalvanize*.

	predictor	model 1	model 2	model 3	model 4
	variables	(*β*)	(*β*)	(*β*)	(*β*)
demographics	age	−0.203***	−0.200***	−0.129***	−0.095*
	gender	0.079*	0.079*	0.059	0.069
	S.E. group	0.022	0.023	0.037	0.035
experimental group	endorse moral hazard versus control		−0.070	−0.062	−0.070
	counter moral hazard versus control		−0.087	−0.068	−0.081
environmental beliefs	scepticism score			−0.285***	−0.272***
	env. group membership			0.062	0.054
values	self-enhancing				0.221***
	self-transcending				0.077
adjusted *R*^2^		0.047	0.050	0.130	0.182

***p<0.001, *p<0.05.

## 4. Discussion

The study was designed to examine four research questions which we discuss in turn. First, the moral hazard argument against geoengineering was perceived as more convincing overall than a counter-argument (RQ1). Both arguments suggested that the presence of geoengineering as a policy option would impact on existing levels of mitigation, but the counter-argument proposed this was not cause for concern. From participants’ preference for the moral hazard argument, we can infer that members of the public agree that reducing mitigation efforts would be a negative outcome. This is also supported by existing research [31,32]. The central premise of the moral hazard argument against geoengineering—that the introduction of geoengineering into the policy landscape would have an adverse impact on existing mitigation efforts—is therefore likely to resonate with members of the public.

However, we also found strong evidence that levels of climate change scepticism and participants’ underlying value orientations played an important role in their perceptions of both the persuasiveness of the moral hazard argument and other attitudinal measures (RQ2 and RQ3). In particular, individuals who expressed higher levels of climate change scepticism were less likely to be persuaded by the moral hazard argument. This is a striking finding, suggesting that, although there may be a general favourability towards the logic of the moral hazard argument, its persuasiveness will be moderated by people's existing views about climate change.

Supporting this idea that the moral hazard argument may have a differential impact on different types of people, there were distinct ‘profiles’ for each of the three statements capturing different levels of moral hazard. People who were more self-oriented, more sceptical about climate change and who were of higher socio-economic status were more likely to agree that their *own* behaviour would be impacted by the prospect of geoengineering. As wealth and socio-economic status are major predictors of an individual's carbon footprint [37], this raises the possibility that people who consume more may be more inclined to view geoengineering as a reason to avoid personal-level behavioural changes than people who consume less.

Conversely, people who scored more highly on self-transcendent values (e.g. concern for the welfare of others) were more likely to perceive *other people* and politicians to be susceptible to the moral hazard trap (although not themselves). Climate sceptics were less concerned about this possibility: for those who are sceptical about climate change, the ‘risk’ of others reducing their motivation to mitigate climate change may in fact not be a ‘risk’ at all, but a step in an appropriate direction.

Climate change sceptics were also significantly *less* likely to agree with the *galvanize* statement (RQ4), suggesting that—at least when materials are presented in a brief written format such as this—learning about geoengineering does not galvanize interest in climate change for this audience. However, those scoring highly on the self-enhancing values scale were more likely to agree that learning about geoengineering made the risks of climate change appear *worse* than they had previously thought. The divergence between the endorsement of self-enhancement values (such as power and wealth) and climate change scepticism here is of considerable interest, as self-enhancing values are widely regarded as underpinning sceptical beliefs about climate change [26]. These findings suggest that, while the prospect of geoengineering may not reduce scepticism about climate change *per se*, it may still represent a climate policy that appeals to self-enhancing values more effectively than more conventional policies (a conclusion supported by [29]. It is also worth reflecting on the relatively weak level of coherence of the four items used to measure ‘self-enhancing’ values. With a stronger set of items, the scale might have had even greater predictive value.

The data obtained shed light on some important practical questions about whether—and for which groups of the public—moral hazard arguments about geoengineering are likely to play an important role. On the one hand, those sceptical about climate change are both less persuaded by a moral hazard argument against climate change and more likely to report that they would be ‘susceptible’ to the trap of a personal-level moral hazard. On the other hand, we found evidence that geoengineering may represent a climate policy that sits more comfortably with ‘self-enhancing’ value orientations (a predictor of climate change scepticism). Thus, it would appear that there is an ambiguous relationship between climate change scepticism and perceptions of geoengineering: it may simultaneously engage sceptics in the prospect of tackling climate change while lessening their inclination to engage in personal-level actions.

There are also some limitations of this study. First, despite a reasonably large sample, the differences obtained in terms of mean judgements and the variance accounted for in the regression analyses were relatively modest. The experimental manipulation itself had a significant impact on only some of the variables we measured (although there was a clear and significant effect on ratings of the convincingness of the opposing arguments). This suggests that the materials constructed for the study did not evoke strong reactions from participants. More interactive materials—perhaps involving video presentations or explanatory graphics—may have enhanced their effectiveness. Support for geoengineering was not significantly altered by the experimental manipulation at all, and, in line with previous literature, people with higher and lower levels of scepticism responded to the argument in different ways [38]. This suggests that the overall impact of moral hazard arguments in public and policy discourse may be simply to reinforce existing beliefs. For those strongly opposed to geoengineering, the moral hazard argument presents another piece of evidence confirming their views; for those sceptical about climate change, the moral ‘hazard’ of reducing mitigation is in fact not a hazard at all.

In addition, it is clear that the *counter moral hazard* argument could have been constructed differently. In this study, the argument states that, although geoengineering may distract from current policies, this is not in fact a moral hazard. An alternative—and arguably just as plausible—counter-argument would be that, although reducing current commitments on mitigation would be a negative outcome, geoengineering will not in fact cause this to happen. In other words, geoengineering will coexist with existing policies, in the same way that adaptation now coexists with mitigation.

However, while there are undoubtedly multiple ways of conceiving of the moral hazard argument, the contribution of this study is in beginning to quantify what public responses to these may be. As geoengineering continues to rise up the science and policy agenda, the current results provide an indication of how compelling the moral hazard argument is likely to be, but also what *sorts* of people may be persuaded by it.

Here, as with so many other areas of the debate about climate change policies, personal values and levels of scepticism are likely to play a crucial role. This study provides the first quantitative evidence that moral hazard arguments about geoengineering may be differentially persuasive to different types of people. The key conclusion is that for people who are sceptical about climate change, wealthier and more self-oriented, the prospect of geoengineering may reduce their own motivation to engage in sustainable behaviour.

## Supplementary Material

Appendices_Electronic Supplementary Materials

## Footnotes

1 This figure is somewhat higher than those obtained in some other national surveys. Typically, between 3% and 5% of the population state membership of an environmental organisation (e.g. British Household Panel Survey, https://www.iser.essex.ac.uk/bhps/documentation/volb/wave5/eindresp9.html#EORGMC).

2 Voting intention is not included in this or further regressions. Preliminary analyses established no significant relationships between voting intention and views on geoengineering. Given the space required to report multiple dummy-coded comparisons in the regression table, we opted to exclude them altogether. A similar decision was made with regard to reported knowledge about geoengineering: preliminary correlational analyses indicated that knowledge did not correlate with judgements about geoengineering in general or the moral hazard variables. With such a low level of knowledge (and by extension very limited variation), we did not include this variable in regression analyses.
