# Supplementary material for: Geoengineering, climate change scepticism and the ‘moral hazard’ argument: an experimental study of UK public perceptions
Source: Philos Trans A Math Phys Eng Sci. 2014 Dec 28;372(2031):20140063. doi: 10.1098/rsta.2014.0063 (PMC4240956; doi:10.1098/rsta.2014.0063)
Supplement: Appendices_Electronic Supplementary Materials [file rsta20140063supp1.docx]

**Electronic Supplementary Material**

**Appendix 1:** Scepticism scale (Whitmarsh, 2011) and value items (from Schwartz, 1992)

*Scepticism scale (Whitmarsh, 2011)*

1. Claims about climate change are exaggerated

2. I do not believe climate change is a real problem

3. I am uncertain about whether climate change is really happening

4. The evidence for climate change is unreliable

5. Too much fuss is made about climate change

6. The media is often too alarmist about issues like climate change

7. I am certain climate change is happening

8. There is solid evidence of climate change

9. Climate change is something that frightens me

10. Climate change is something I worry about

11. I am concerned about climate change

12. The effects of climate change will be catastrophic

*Items from Schwartz’ (1992) values inventory*

*Self-enhancing values*

1. SOCIAL POWER (control over others, dominance)

2. WEALTH (material possessions, money)

3. SOCIAL RECOGNITION (respect, approval by others)

4. AUTHORITY (the right to lead or command)

Self-transcending values

5. EQUALITY (equal opportunity for all)

6. INNER HARMONY (at peace with myself)

7. A WORLD AT PEACE (free of war and conflict)

8. SOCIAL JUSTICE (correcting injustice, care for the weak)

Appendix 2: Factsheets used in the study

*ClimateInfo.org.uk*

*Factsheet #027*

Geoengineering: A new approach for tackling climate change?

The term ‘climate change’ is used to describe the effect that higher levels of carbon dioxide are having on the planet. Carbon dioxide traps heat, and so the more that we release, through burning coal, oil and gas, and by cutting down trees which absorb carbon dioxide, the warmer the world will become. As the world gets warmer, scientists predict that there will be range of serious risks, including droughts and heat waves cause by higher temperatures, rising sea-levels threatening low-lying areas, disruption to food supply chains and more extreme and unpredictable flooding.

The kinds of responses that currently exist for tackling climate change include:

• Shifting to renewable sources of energy (e.g. wind or solar), or using more nuclear power

• Reducing the amount of energy that we use (e.g. through big changes in our lifestyles)

• Introducing green taxes, and regulations on industry to be more efficient and waste less energy

There is one other set of ideas, called ‘geoengineering’. This is the term used to describe technologies that could, in the future, be used to intentionally manipulate the Earth’s climate to combat the effects of climate change. There are two ways that geoengineering could be used – either by removing carbon dioxide from the atmosphere and storing it elsewhere, or by reflecting a certain amount of sunlight back into space.

Most geoengineering technologies have not yet been developed, and so there is a great deal of uncertainty about their risks and benefits. There are likely to be some serious risks and side effects associated with geoengineering (e.g. changes to global rainfall patterns) but they are not yet well understood.

Professor North, a leading Atmospheric Physicist, explains it like this:

(*EXPERIMENTALLY MANIPULATED TEXT HERE – SEE METHODS SECTION OF MANUSCRIPT)*
